# Incentives and constraints: An optimization mechanism for improving the performance of farmland protection entities

**DOI:** 10.1371/journal.pone.0320750

**Published:** 2025-06-02

**Authors:** Zeyuan Li, Xiaoying Zhao

**Affiliations:** 1 School of Maxism, Beijing Forestry University, Beijing, China; 2 Center for Green Development and Rural Land Research in China, Beijing, China; 3 School of Finance and Economics, Shandong University of Science and Technology, Tai’an, China; PLOS ONE, UNITED KINGDOM OF GREAT BRITAIN AND NORTHERN IRELAND

## Abstract

Farmland is the most crucial resource for food production, and implementing farmland protection is a long-term strategy to achieve the sustainable use of this vital resource. Farmland protection is not only a priority for policymakers but also requires the cooperation of management departments and policy implementation bodies. Within China’s “county-township-village” farmland protection organizational structure, the current pressing issue is how to align the performance of grassroots governments using an incentive-constraint system and guide village-level cooperation to effectively implement farmland protection. This paper establishes a tripartite evolutionary game model focused on the incentive-constraint system for farmland protection, analyzing the demands and preferences of stakeholders and examining the strategic choices under the influence of multiple factors, such as farmland protection benefits, collusion behavior, accountability, financial support, and incentive-constraint standards. The optimal strategy combination for the tripartite game is discussed. The study found the following: (1) Village collectives tend to adopt active farmland protection strategies regardless of whether township governments provide financial support. (2) The increase in administrative rewards and punishments weakens the concealment behavior of township governments, becoming more effective as the success rate of assessments increases. (3) In areas with a high success rate of assessments, administrative incentives effectively improve stakeholder behavior; in areas with a low success rate, a higher level of incentive and constraint combinations can improve multi-party cooperation. (4) Administrative incentives for township governments must not fall below the administrative costs of strict supervision. Therefore, this paper proposes an optimization strategy for coordinating stakeholders in farmland protection in China. It suggests that county governments should strictly implement supervision and assessment, prioritize the use of incentive mechanisms to enhance the transparency of township government supervision, and encourage village collectives to develop a self-consciousness and behavior conducive to farmland protection.

## 1. Introduction

As the primary carrier of grain production [1], farmland protection and rational utilization have long been focal issues for developing countries [2–6]. China sustains 22% of the global population with less than 10% of the world’s farmland [7]. A well-established land protection system is particularly crucial for China, especially for its rural poor population [8–10]. “Protection through utilization” has been a key strategy in China’s farmland protection efforts and remains a cornerstone of its restrictive policies [11,12]. As a scarce resource with the attributes of a public good, farmland is difficult for private individuals to monopolize, and it is challenging to secure payment from beneficiaries. The nature of land ownership in rural China means that farmers only have usage rights, not ownership rights. The government plays a dominant role in farmland protection. Additionally, farmers often lack awareness of the societal and ecological benefits provided by farmland, which hampers the creation of a proactive societal environment for farmland protection. Therefore, it is essential to provide economic, policy, and land-use incentives to those responsible for conservation, based on their actual effectiveness in maintaining land sustainability and ecological functions. An effective voluntary protection mechanism can only be realized when the private benefits of farmland protection exceed the opportunity costs. More developed agricultural countries such as the United States, Germany, and Japan, as well as developing countries like India, regulate farmers’ land use through agricultural subsidy policies [13–15]. While continuously improving the restrictive ‘hard measures’ for farmland protection, China is increasingly focusing on strengthening positive incentives for farmland conservation, as shown in Table 1. However, the current assessment system primarily evaluates the performance of local policy implementation, neglecting the incentives and constraints for grassroots governments themselves. This significantly reduces the efficiency of farmland protection.

**Table 1:** Main components of the farmland protection incentive and constraint system at the national level in China.

Policy Year	Policy Content	Attributes
1997	Balance the occupation and replenishment of farmland	Constraints
2006	First proposed the “18 billion mu of farmland line must be preserved”	Constraints
2007	Strictly prevent and correct unauthorized agricultural land conversion	Constraints
2008	Permanent farmland and establish a protection and compensation mechanism	Incentives
2009	Enforce the strictest farmland protection and land conservation systems	Constraints
2012	Ensure the farmland stock remains at 18.18 billion mu	Constraints
	Launch farmland protection compensation pilot program	Incentives
2016	Improve the farmland protection compensation system	Incentives
2017	Improve the farmland protection compensation mechanism	Incentives
2020	Strictly guard the 18 billion mu of farmland line	Constraints
2022	National farmland stock shall not be less than 1.865 billion mu	Constraints
2023	Provide economic incentives to provinces that exceed farmland protection targets	Incentives

Strictly maintaining the farmland “red line” and stabilizing grain output is not only a fundamental guarantee for China to achieve food security but also relates to the overall development and security of the nation [16]. In 2006, China first introduced the binding target of 1.8 billion mu for farmland, known as the “red line”. By the end of 2019, the total area of farmland in China reached 1.918 billion mu. However, compared to 2009, China lost 113 million mu of farmland over the subsequent decade, gradually approaching the “red line”. Since 2021, China has experienced three consecutive years of growth in farmland, with a net increase of 17.58 million mu, effectively halting the long-standing decline in farmland. Various regions have also launched multiple pilot programs and model explorations. For instance, Anhui and Hubei provinces have implemented the approach of small fields merging into larger fields, where leveled contiguous land is collectively transferred to large-scale farmers or companies for management. In addition, Zhanqi Village in Sichuan has conducted comprehensive land consolidation, with villagers relocating to compact communities, leaving approximately 68 acres of land reserved for agricultural cultivation [17]. However, China is about to initiate a second round of contract extensions for 30 years, meaning that the basic rural management system of collective land ownership will remain unchanged for the foreseeable future. It is important to recognize that China has limited per capita farmland, overall low land quality, insufficient reserve land resources, and a continued issue of fragmented and scattered farmland. Agriculture in China remains a labor-intensive industry, facing realistic challenges such as an aging population, declining labor quality, rural-to-urban migration, and low grain subsidies for farmers, all of which pose significant difficulties for land protection [18,19]. Moreover, the stock of high-quality farmland does not align with the designated target of 1.546 billion mu of permanent basic farmland, highlighting the urgent need to optimize the mechanism for improving farmland protection performance [20].

Globally, farmland protection typically requires governments intervention through legal means [21], market instruments [22], and planning measures [23–25]. To achieve the goal of farmland protection, the central government capitalizes on the characteristics of a pressure-based system, by breaking down various directive indicators into political tasks assigned to each province. Provincial governments then further decompose these tasks in a top-down manner, passing them down step by step. Ultimately, the responsibility for implementing farmland protection lies with township governments and village collectives, forming a hierarchical organizational structure of “county-township-village” for farmland protection. County governments act as the principals, with village collectives serving as agents for farmland protection, while township governments oversee the process. Simultaneously, township governments report their performance in implementation to county governments, thus creating a dual ‘principal-agent’ relationship for farmland protection, with township governments serving as intermediaries. This organizational structure can lead to a mismatch between the interests and goals of the subjects involved and the governing ideas. However, the official performance appraisal (OPA) has always been key factor in the promotion of Chinese officials [26]. When the economic and political benefits of farmland protection are not met, township governments may engage in opportunistic behavior to mitigate unfavorable conditions, leading to a deviation from the intended farmland protection outcomes [27]. If village collectives do not receive adequate financial support, there is a high likelihood of moral hazard and adverse selection behaviors [28,29]. Ultimately, higher-level governments may be unable to fully and timely monitor or verify the true performance of farmland protection [30,31]. In such cases, higher-level governments risk falling into the “Tacitus Trap,” facing a loss of credibility and depletion of public resources. Therefore, optimizing the farmland protection assessment mechanism requires balancing the interests of governments at all levels with the overall policy effectiveness [32].

Some scholars have examined the incentive effects of administrative measures on local officials, arguing that incentives closely tied to their promotion may better contribute to societal stability and economic growth [33,34]. As part of national governance, local governments’ decisions have a significant impact on the implementation of farmland protection policies and environmental outcomes [35]. Research shows that promotion incentive mechanisms notably influence local governments’ policy choices [36], with officials who meet environmental performance targets being more likely to be promoted [37–40]. The central government establishes political objectives and uses promotion as a political reward to foster a competitive and accountable incentive system for local officials [41].

Additionally, existing studies have explored the regulatory mechanisms between government departments. Some scholars have discussed the top-down administrative supervision mechanism, where higher-level governments communicate policy requirements to grassroots governments, which then implement them based on local conditions [42]. This model is believed to effectively enhance the policy implementation capacity of local governments [43,44]. Particularly within China’s highly centralized political system, the central government exercises vertical control over local governments, including the appointment and promotion of local officials, which inherently motivates these officials to prioritize rapid economic growth in their jurisdictions [45,46]. Farmland protection programs in the United States include converting agricultural land for urban use, establishing voluntary agricultural zones, and strengthening tax policies. In North America, several farmland protection laws have been enacted to restrict further residential or industrial development in designated areas [47,48]. In France, the conversion of farmland to urban use is primarily regulated by municipal authorities through binding zoning plans, while Italy has undergone a decentralization process in land use planning [49]. However, some scholars have raised concerns about the effectiveness of governmental oversight. In Montpellier, France, extensive debates have been held regarding farmland protection, particularly concerning public actors and farmer representatives, with criticism that the policies have overlooked societal justice, creating new inequalities between farmers and other residents [50]. For instance, Shen et al. argue that the unification of policy execution and supervision at the grassroots level creates opportunities for manipulation of environmental and quality targets [51], particularly in the illegal conversion of agricultural land [52], posing significant challenges to societal stability and the ecological environment. Under the influence of decentralization in China, local governments have long pursued economic development at the expense of the environment to win political promotions and gain financial benefits [53–55]. Although governments attract capital by lowering tax rates, profit- driven behavior among local governments can lead to government failure, resulting in reduced public revenues [56]. Fiscal competition within governments can also lead to inefficiencies in the scale and structure of local public revenues, thereby diminishing public service expenditures that benefit residents.

In this context, this study constructs a tripartite game model involving county governments, township governments, and village collectives to explore the role of the assessment mechanism in improving farmland protection performance. The study also analyzes the applicability of administrative incentives and penalties, along with the criteria for rewards and punishments, aiming to identify strategies that foster a win-win outcome for all three parties.

## 2. Evolutionary game model

### 2.1 Problem description

**Fig 1:**
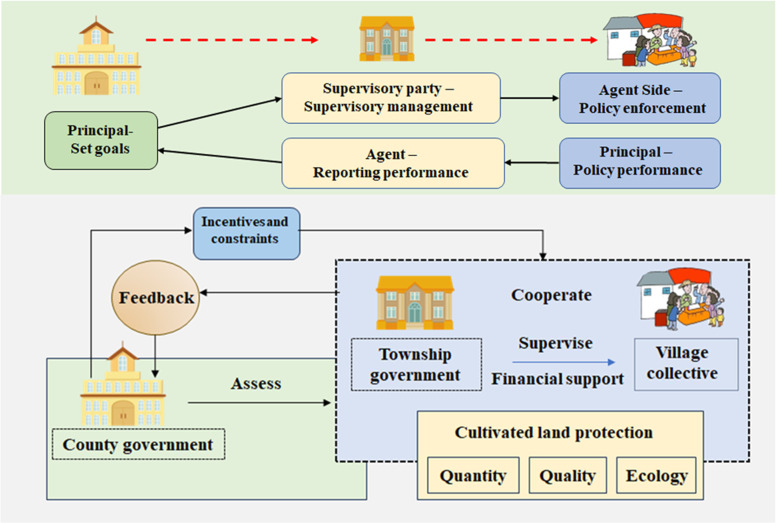
Schematic diagram of the “county-township-village” three-level farmland protection incentive-constraint mechanism.

China has established a hierarchical organizational structure for farmland protection, wherein township governments and village collectives maintain a principal-agent relationship. Strict supervision is necessary to ensure that village collectives fulfill their farmland protection responsibilities. However, instances may arise where the actual performance of village collectives is concealed, including issues such as illegal farmland use and unauthorized farmland occupation. While village collectives may make long-term efforts to fulfill their duties and implement required measures, challenges such as insufficient funding or managerial negligence can lead to failures in meeting responsibilities. In such cases, village collectives may attempt to conceal their shortcomings, often with the assistance of township governments, to pass performance assessments. From an administrative management perspective, if township governments engage in such concealment, county governments hold the authority to take corrective actions against the responsible department staff. Changes in positions and salary adjustments can serve as direct tools of incentives or constraints. As long as an assessment system is in place, it inevitably involves rewards for high performers and penalties for violators. This study advocates strengthening administrative incentives and constraints mechanisms to enhance the supervisory capacity of township governments, ensure transparent regulation, and encourage village collectives to actively implement farmland protection. Ultimately, this approach aims to institutionalize the enforcement of grassroots farmland protection through administrative incentives. The logical relationships in the “county-township-village” farmland protection evolutionary game model are illustrated in Fig 1.

### 2.2 Basic assumptions

To analyze the strategy choices, equilibrium stability, and evolutionary process of each party, and to comprehensively analyze the multiple factors in the model, the following assumptions are made:

** Assumption 1**. County governments, township governments, and village collectives are all bounded rational participants who continuously adjust their strategy choices over time to maximize their benefits.
** Assumption 2.** The decision-making approach of county governments in implementing farmland protection policies differs from that of township and village collectives. Over time and with the progress of objectives, the strategy may be adjusted. It is assumed that county governments will assess the completion of grassroots farmland protection tasks with a probability of x(0≤x≤1), while the probability of not conducting assessments is 1−x. It is assumed that township governments will conceal the passive performance of village collectives with a probability of y(0≤y≤1), while the probability of transparent supervision is 1−y. It is assumed that the village collectives choose passive performance with a probability of z(0≤z≤1), while the probability of active performance is 1−z.
** Assumption 3.** Regardless of whether the village collectives strictly implement farmland protection work, township governments, as the higher-level financial department, are responsible for assisting with the funding applications for farmland protection submitted by the village level.

### 2.3 Parameter settings

The municipal government is responsible for achieving the comprehensive benefits of farmland protection, denoted as $r_{1}$, and ensuring governance credibility, represented by g. It also supervises the assessment of grassroots land protection responsibilities based on established evaluation criteria. Despite the municipal government’s implementation of a stringent evaluation and incentive system to encourage township governments and village collectives to uphold land protection, instances of “collusion” between these entities to report false achievements have been observed. Therefore, it is assumed that the probability of successful policy implementation assessment by county governments is p. If the concealment actions of township governments or the passive performance of village collectives are not detected, the assessment by county governments will fail. In this case, the credibility of county governments’ credibility is $l_{1}$, and the societal loss is d where $l_{1}<g$. The direct cost of county governments to assess township governments is $c_{1}$, and since the governments seek to achieve both performance and credibility, it is assumed that $c_{1}<l_{1}<g$.

Township governments monitor the implementation of farmland protection by village collectives in real time and report annually to county governments. It is assumed that the level of supervision and implementation between township governments and village collectives is characterized by perfect information symmetry, while there is incomplete information symmetry between township governments and county governments. Given that the current farmland subsidy policy only includes rewards and lacks a deterrent effect on grassroots entities, it contradicts the objectives of both incentives and constraints. Therefore, it would be more scientifically rigorous to incorporate both rewards and penalties into the game theory model. If a township government chooses to implement strict and transparent supervision, the administrative cost it faces is $c_{2}$, while the administrative rewards it can receive include positive incentives such as promotions or public recognition, denoted as $r_{2}$. If the township government chooses to abuse its power for personal gain by concealing the negative actions of village collectives, the administrative cost it faces will be $c_{3}$, where $c_{3}<c_{2}$. Additionally, if the township government’s concealment is discovered, administrative penalties will be imposed to curb such behavior, such as demotion or salary deductions. In this case, the administrative loss incurred is $l_{2}$, which includes the loss of administrative rewards $r_{2}$, diminished public trust, and damage to personal reputation, among others. It follows that $l_{2}>r_{2}$.

Village collectives receive financial support funds for farmland protection subsidized by township governments, denoted as s. It is assumed that township governments are the primary bearers of the implementation costs for grassroots farmland protection and only delegate specific tasks to village collectives for execution. When the performance is fully achieved, the village level can obtain the direct comprehensive benefits of farmland protection, denoted as $r_{3}$. At this time, the input cost of farmland protection is $c_{4}$. If the village collectives do not comply with regulations and use the funds for other investments, it is assumed that no policy benefits will be generated, but speculative gains will be obtained, denoted as $r_{4}$. At this time, the input cost is$c_{5}(c_{5}<c_{4}$), and the accountability loss $l_{3}$ is directly recovered by township governments. If village collectives attempt to collude with higher authorities, the collusion cost for seeking government protection is denoted as $c_{6}$. It is known that $c_{6}<l_{3}$, and $c_{6}<c_{4}$. It is assumed that if village collectives actively perform their duties and government supervision at all levels is fully effective, collusion will not occur. Table 2 presents the relevant variables and their meanings regarding the interests and demands of the game participants.

**Table 2:** Related variables and meanings of the interests of game participants.

Variable	Description
$c_{1}$	The assessment cost of county governments
$l_{1}$	The credibility of county governments in the case of dereliction of duty
d	societal losses caused by the negative implementation of farmland protection
$r_{1}$	Comprehensive benefit of county governments farmland protection
g	The credibility of county governments under strict assessment
p	The probability of successful policy implementation assessment
$c_{2}$	Township governments under strict supervision of administrative costs
$c_{3}$	Township governments conceal the actual administrative costs
$l_{2}$	The administrative penalty for township governments upon discovery of concealment
$r_{2}$	Administrative incentives received by township governments
$c_{4}$	The administrative cost of village collective under active responsibility
$c_{5}$	The administrative cost of village collective under negative responsibility
$c_{6}$	Rent-seeking costs of village collective
$l_{3}$	The accountability loss for village collectives when negative performance is revealed
$r_{3}$	Comprehensive benefits of village collective when the goal is completed
s	Financial support from the township to the village collective
$r_{4}$	The profit of village collective speculation

### 2.4 Game payoff matrix

County governments may monitor and measure farmland protection and farmland loss in order to grasp the current situation of farmland. It may also gradually cancel the monitoring and measurement of farmland with the change of system. Therefore, the county government’s strategic options are either to conduct assessments or not. The township government’s strategic choices are to either conceal the truth or ensure transparent supervision, while the village collective’s strategies are to either neglect responsibilities or actively fulfill them. This results in a total of eight possible strategic combinations among the three parties. The payoff matrix for these combinations is shown in Table 3.

**Table 3:** Payoff matrix of the three game participants.

Strategies		Township Conceals Truth		Township Supervises Transparently	
		Village Collectives Perform Passively	Village Collectives Perform Actively	Village Collectives Perform Passively	Village Collectives Perform Actively
**County government**	Assess	$\left[\begin{array}{c} pg+(1-p)l_{1}-d-c_{1}\\ (1-p)r_{2}+c_{6}+pl_{3}-c_{3}-pl_{2}-s\\ s+r_{4}-c_{5}-pl_{3}-c_{6} \end{array}\right]$	$\left[\begin{array}{c} r_{1}+pg+(1-p)l_{1}-c_{1}\\ (1-p)r_{2}-c_{3}-pl_{2}-s\\ s+r_{3}-c_{4} \end{array}\right]$	$\left[\begin{array}{c} g-d-c_{1}\\ l_{3}+r_{2}-c_{2}-s\\ s+r_{4}-c_{5}-l_{3} \end{array}\right]$	$\left[\begin{array}{c} r_{1}+g-c_{1}\\ r_{2}-c_{2}-s\\ s+r_{3}-c_{4} \end{array}\right]$
	Not Assess	$\left[\begin{array}{c} -d-l_{1}\\ c_{6}-c_{3}-s\\ s+r_{4}-c_{5}-c_{6} \end{array}\right]$	$\left[\begin{array}{c} r_{1}-l_{1}\\ -c_{3}-s\\ s+r_{3}-c_{4} \end{array}\right]$	$\left[\begin{array}{c} -d-l_{1}\\ l_{3}-c_{2}-s\\ s+r_{4}-c_{5}-l_{3} \end{array}\right]$	$\left[\begin{array}{c} r_{1}-l_{1}\\ -c_{2}-s\\ s+r_{3}-c_{4} \end{array}\right]$

## 3. Construction and analysis of the evolutionary game model

### 3.1 County governments strategy selection and equilibrium analysis

Assuming the expected payoff for county governments adopting the assessment strategy is $E_{m1}$, and the expected payoff for adopting the non-assessment strategy is $E_{m2}$, the average expected payoff for county governments’ strategy choice is $E_{m}$, then:


$$E_{m1}=yz\left[pg+(1-p)l_{1}-d-c_{1}\right]+y(1-z)\left[r_{1}+pg+(1-p)l_{1}-c_{1}\right]+(1-y)z\left(g-d-c_{1}\right)+(1-y)(1-z)\left(r_{1}+g-c_{1}\right)$$ (1)

$$E_{m2}=yz\left(-d-l_{1}\right)+y(1-z)\left(r_{1}-l_{1}\right)+(1-y)z\left(-d-l_{1}\right)+(1-y)(1-z)\left(r_{1}-l_{1}\right)$$ (2)

$$E_{m}=xE_{m1}+(1-x)E_{m2}$$ (3)

The dynamic equation expression for the assessment strategy taken by county governments is as follows:


$$F(x)=\frac{dx}{dt}=x\left(E_{m1}-E_{m}\right)=x(1-x)\left(E_{m1}-E_{m2}\right)=x(1-x)\left(ypg+yl_{1}-ypl_{1}+g-c_{1}-yg+l_{1}\right)$$ (4)

According to the stability theorem of differential equations, the evolutionary stable condition of county governments’ assessment strategy should satisfy: F(x)=0 and $\frac{dF(x)}{dx}<0$.

Given:


$$\frac{dF(x)}{dx}=(1-2x)\left(ypg+yl_{1}-ypl_{1}+g-c_{1}-yg+l_{1}\right)$$ (5)

Let:


$$G(y)=ypg+yl_{1}-ypl_{1}+g-c_{1}-yg+l_{1}$$ (6)

$Since\frac{\partial G(y)}{\partial y}<0alwaysholds,G(yisadecreasingfunction.$ The solutions to F(x)=0 are x=0or x=1 or $y^{*}=\frac{c_{1}-g-l_{1}}{pg+l_{1}-pl_{1}-g}$. When $y=y^{*}$, $\frac{dF(x)}{dx}\equiv 0,$at this point, county governments cannot determine a stable strategy. When $y<y^{*}$, $G(y)>0,atthispoint,\frac{dF(x)}{dx}{|}_{x=0}>0,$ $\frac{dF(x)}{dx}{|}_{x=1}<0$ and x=1is the evolutionary stable strategy of county governments. This implies that township governments strictly supervise and truthfully report the fulfillment of village collectives’ farmland protection responsibilities, and county governments adopt the assessment mechanism for farmland protection, forming a positive promotion state for farmland protection. Conversely, when $y>y^{*}$, $G(y)<0,inwhichcase\frac{dF(x)}{dx}{|}_{x=0}<0$, $\frac{dF(x)}{dx}{|}_{x=1}>0$ and x=0 is the evolutionary stable strategy of county governments, indicating a higher probability of concealing the actual performance of village collectives. As a result, the county government will not adopt the assessment strategy.

### 3.2 Township governments strategy selection

Assuming the expected benefit of township governments choosing the strategy of concealing the truth is $E_{n1}$, and the expected benefit of choosing the strategy of transparent supervision is $E_{n2}$, the average expected benefit of township governments’ strategy is$E_{n}$, then:


$$E_{n1}=xz\left[(1-p)r_{2}+c_{6}+pl_{3}-c_{3}-pl_{2}-s\right]+x(1-z)\left[(1-p)r_{2}-c_{3}-pl_{2}-s\right]+(1-x)z\left(c_{6}-c_{3}-s\right)+(1-x)(1-z)\left(-c_{3}-s\right)$$ (7)

$$E_{n2}=xz\left(l_{3}+r_{2}-c_{2}-s\right)+x(1-z)\left(r_{2}-c_{2}-s\right)+(1-x)z\left(l_{3}-c_{2}-s\right)+(1-x)(1-z)\left(-c_{2}-s\right)$$ (8)

$$E_{n}=yE_{n1}+(1-y)E_{n2}$$ (9)

The dynamic equation expression for township governments’ strategy of concealing the truth is as follows:


$$F(y)=\frac{dy}{dt}=y\left(E_{n1}-E_{n}\right)=y(1-y)\left(E_{n1}-E_{n2}\right)=y(1-y)\left(xzpl_{3}-xpr_{2}-xpl_{2}+zc_{6}-c_{3}-zl_{3}+c_{2}\right)(10)$$ (10)

According to the stability theorem of differential equations, the evolutionary stable condition of township governments’ strategy of concealing the truth should satisfy: F(y)=0 and $\frac{dF(y)}{dy}<0$.

Given:


$$\frac{dF(y)}{dy}=(1-2y)\left(xzpl_{3}-xpr_{2}-xpl_{2}+zc_{6}-c_{3}-zl_{3}+c_{2}\right)$$ (11)

Let:


$$H(x,z)=xzpl_{3}-xpr_{2}-xpl_{2}+zc_{6}-c_{3}-zl_{3}+c_{2}$$ (12)

$Since\frac{\partial H(x,z)}{\partial z}<0alwaysholds,H(x,zisadecreasingfunction.ThesolutionstoF(y)=0arey=0ory=1orz^{*}=\frac{xpr_{2}+xpl_{2}+c_{3}-c_{2}}{xpl_{3}+c_{6}-c_{3}}$. When $z=z^{*}$, $\frac{dF(y)}{dy}\equiv 0$, at this point, township governments cannot determine a stable strategy. When $z<z^{*}$, $H(x,z)>0,atthispoint,\frac{dF(y)}{dy}{|}_{y=0}>0$, $\frac{dF(y)}{dy}{|}_{y=1}<0$ and y=1 is the evolutionary stable strategy of township governments. Township governments may still try to “collude” with the village collective in order to maximize the profit and conceal the actual situation of the policy implementation.

Similarly, when $z>z^{*}$, $H(x,z)<0,inwhichcase\frac{dF(y)}{dy}{|}_{y=0}<0$, $\frac{dF(y)}{dy}{|}_{y=1}>0$ and y=0 is the evolutionary stable strategy of township governments. This implies that when the probability of village collectives passively fulfilling responsibilities is higher, township governments will consciously improve supervision awareness of village collectives’ farmland protection responsibilities to avoid concealment behavior being discovered by higher authorities and tend to choose a “transparent” supervision strategy.

### 3.3 Village collective strategy selection

Assuming the expected benefit of the village collective choosing the strategy of passive fulfillment is $E_{u1}$, and the expected benefit of choosing the strategy of active fulfillment is $E_{u2}$, the average expected benefit of the village collective’s strategy is $E_{u}$, then:


$$E_{u1}=xy\left(s+r_{4}-c_{5}-pl_{3}-c_{6}\right)+x(1-y)\left(s+r_{4}-c_{5}-l_{3}\right)+(1-x)y\left(s+r_{4}-c_{5}-c_{6}\right)+(1-x)(1-y)\left(s+r_{4}-c_{5}-l_{3}\right)$$ (13)

$$E_{u2}=xy\left(s+r_{3}-c_{4}\right)+x(1-y)\left(s+r_{3}-c_{4}\right)+(1-x)y\left(s+r_{3}-c_{4}\right)+(1-x)(1-y)\left(s+r_{3}-c_{4}\right)$$ (14)

$$E_{u}=zE_{u1}+(1-z)E_{u2}$$ (15)

The dynamic equation expression for the village collective’s strategy of passive fulfillment is as follows:


$$F(z)=\frac{dz}{dt}=z\left(E_{u1}-E_{u}\right)=z(1-z)\left(E_{u1}-E_{u2}\right)=z(1-z)\left(r_{4}-xypl_{3}-yc_{6}-c_{5}-l_{3}+yl_{3}-r_{3}+c_{4}\right)$$ (16)

According to the stability theorem of differential equations, the evolutionary stable condition of the village collective’s strategy of passive fulfillment should satisfy: F(z)=0 and $\frac{dF(z)}{dz}<0$.

Given:


$$\frac{dF(z)}{dz}=(1-2z)\left(r_{4}-xypl_{3}-yc_{6}-c_{5}-l_{3}+yl_{3}-r_{3}+c_{4}\right)$$ (17)

Let:


$$T(x,y)=r_{4}-xypl_{3}-yc_{6}-c_{5}-l_{3}+yl_{3}-r_{3}+c_{4}$$ (18)

Since $\frac{\partial T(x,y)}{\partial x}<0$ always holds. ThesolutionstoF(z)=0 are $z=0orz=1or=\frac{r_{4}-yc_{6}-c_{5}-l_{3}-r_{3}+c_{4}}{ypl_{3}}$. When $x=x^{*}$, $\frac{dF(z)}{dz}\equiv 0$, in which case the village collective cannot determine a stable strategy. When $x<x^{*}$, T(x,y)>0, $the\frac{dF(z)}{dz}{|}_{z=0}>0$, $\frac{dF(z)}{dz}{|}_{z=1}<0$, z=1 is the evolutionary stable strategy of the village collective, indicating that when the assessment intensity of county governments is low, it is not conducive to stimulating the enthusiasm of village collectives for farmland protection. Similarly, when $x>x^{*}$, T(x,y)<0, the $\frac{dF(z)}{dz}{|}_{z=0}<0$, $\frac{dF(z)}{dz}{|}_{z=1}>0$, z=0 is the evolutionary stable strategy of the village collectives, meaning that when the county governments’ level of farmland protection assessment is high, village collectives are more inclined to adopt an active farmland protection strategy.

The three replicated dynamic equation is shown below:


$$\begin{array}{c} \left\{ \;\;\;\;\begin{array}{c} \frac{dx}{dt}=x(1-x)\left(ypg+yl_{1}-ypl_{1}+g-c_{1}-yg+l_{1}\right)\\ \frac{dy}{dt}=y(1-y)\left(xzpl_{3}-xpr_{2}-xpl_{2}+zc_{6}-c_{3}-zl_{3}+c_{2}\right)\\ \frac{dz}{dt}=z(1-z)\left(r_{4}-xypl_{3}-yc_{6}-c_{5}-l_{3}+yl_{3}-r_{3}+c_{4}\right) \end{array}\right. \end{array}$$ (19)

### 3.4 Stability analysis of tripartite evolutionary game equilibrium points

According to the research by Ritzberger and Weibull [57], in the dynamic replication system of a tripartite evolutionary game, the sufficient and necessary condition for evolution to reach the equilibrium point *M* (*x, y, z*)is that *M* (*x, y, z*)is a strict Nash equilibrium. Therefore, this paper considers the asymptotic stability of eight pure strategy equilibrium points of the tripartite principal. From the tripartite dynamic equations, the equilibrium points of the system’s evolutionary game are: $M_{1}(0,0,0)$*、*$M_{2}(1,0,0)$*、*$M_{3}(1,1,0)$*、*$M_{4}(1,0,1)$*、*$M_{5}(0,1,0)$*、*$M_{6}(0,1,1)$*、*$M_{7}(0,0,1)$*、*$M_{8}(1,1,1)$*.*

The evolutionary stable strategies of the differential equation group can be obtained by analyzing the local stability of the Jacobian matrix [58]. The Jacobian matrix of the tripartite evolutionary game system is as follows:


$$\begin{array}{cc} J_{F(x,y,z)}= & (\begin{array}{ccc} \frac{\partial F(x)}{\partial x} & \frac{\partial F(x)}{\partial y} & \frac{\partial F(x)}{\partial z}\\ \frac{\partial F(y)}{\partial x} & \frac{\partial F(y)}{\partial y} & \frac{\partial F(y)}{\partial z}\\ \frac{\partial F(z)}{\partial x} & \frac{\partial F(z)}{\partial y} & \frac{\partial F(z)}{\partial z} \end{array})=\\ & (\begin{array}{ccc} \begin{array}{c} (1-2x)(ypg+yl_{1}-ypl_{1}\\ +g-c_{1}-yg+l_{1}) \end{array} & x(1-x)\left(pg+l_{1}-pl_{1}-g\right) & 0\\ y(1-y)\left(zpl_{3}-pr_{2}-pl_{2}\right) & \begin{array}{c} (1-2y)(xzpl_{3}-xpr_{2}-xpl_{2}\\ +zc_{6}-c_{3}-zl_{3}+c_{2}) \end{array} & y(1-y)\left({xpl_{3}+c}_{6}-l_{3}\right)\\ z(1-z)\left(-ypl_{3}\right) & z(1-z)\left(l_{3}-xpl_{3}-c_{6}\right) & \begin{array}{c} (1-2z)(r_{4}-xypl_{3}-yc_{6}\\ -c_{5}-l_{3}+yl_{3}-r_{3}+c_{4}) \end{array} \end{array}) \end{array}$$ (20)

Using Lyapunov stability theory to determine the stability of the equilibrium points in the evolutionary game, if all eigenvalues of the Jacobian matrix are negative, the equilibrium point of the evolutionary game system will tend towards an asymptotically stable state [59]. Conversely, if there is at least one positive eigenvalue at any equilibrium point of the Jacobian matrix, then that equilibrium point is unstable. Based on this, the stability conditions of the equilibrium points can be obtained, as shown in Table 4. According to the parameter settings, it is known that $\begin{array}{c} C_{2}-C_{3} \end{array}$*>*0, so It can be directly judged that *M*_1_ (0,0,0)is not a stable equilibrium point, indicating that under the condition where county governments do not assess, the strategy combination of township governments supervising transparently and village collectives performing actively is unstable. In that case, what would be the effect if county governments conduct assessments and implement administrative incentives? To explore the impact of the incentive mechanism on improving grassroots farmland protection performance and the influence of major parameter changes on the convergence of strategy choices among different entities, the following deductive analysis is conducted.

**Table 4:** The characteristic value and stability of the equilibrium.

Equant equation	Jacobian matrix eigenvalues	stable condition
*M*_1_ (0,0,0)	*λ*_*11*_$=g-c_{1}+l_{1}$*, λ*_*12*_$=c_{2}-c_{3}$, *λ*_*13*_$=r_{4}-c_{5}-l_{3}-r_{3}+c_{4}$	Instability
*M*_2_ (1,0,0)	*λ*_*21*_$=c_{1}-g-l_{1},$*λ*_*22*_$=-p(r_{2}+l_{2})+c_{2}-c_{3}$, *λ*_*23*_$=r_{4}-c_{5}-l_{3}-r_{3}+c_{4}$	*λ*_*21*_ *<0 λ*_*22*_ *<0* *λ*_*23*_ *<0*
*M*_3_ (1,1,0)	*λ*_*31*_$=-p(g-l_{1})+c_{1}-2l_{1}$*, λ*_*32*_$=p(r_{2}+l_{2})+c_{3}{-c}_{2}$, *λ*_*33*_$=r_{4}-pl_{3}-c_{6}{-c}_{5}-r_{3}+c_{4}$	Instability
*M*_4_ (1,0,1)	*λ*_*41*_$=c_{1}-g-l_{1},$*λ*_*42*_$=p({l_{3}-r}_{2}-l_{2})+c_{6}-c_{3}-l_{3}+c_{2}$, *λ*_*43*_$=-r_{4}+c_{5}+l_{3}{+r}_{3}{-c}_{4}$	Instability
*M*_5_ (0,1,0)	*λ*_*51*_$=p(g-l_{1})-c_{1}+2l_{1}$, *λ*_*52*_$=c_{3}{-c}_{2}$, *λ*_*53*_$=r_{4}-c_{6}{-c}_{5}-r_{3}+c_{4}$	Instability
*M*_6_ (0,1,1)	*λ*_*61*_$=p(g-l_{1})-c_{1}+2l_{1},$*λ*_*62*_$=c_{3}{-c_{6}+l_{3}-c}_{2}$, *λ*_*63*_$={c_{6}{+c}_{5}+r_{3}-r}_{4}-c_{4}$	Instability
*M*_7_ (0,0,1)	*λ*_*71*_$=g-c_{1}+l_{1},$*λ*_*72*_$={c_{6}-c}_{3}{-l_{3}+c}_{2}$, *λ*_*73*_$=c_{5}+l_{3}+r_{3}-r_{4}-c_{4}$	Instability
*M*_8_ (1,1,1)	*λ*_*81*_$=p(l_{1}-g)+c_{1}-2l_{1}$, *λ*_*82*_$=p(l_{2}{{-l}_{3}+r}_{2})-c_{6}+c_{3}+l_{3}-c_{2}$, *λ*_*83*_$=-r_{4}+pl_{3}{+c_{6}{+c}_{5}+r}_{3}{-c}_{4}$	Instability

First, from the assumptions, we can infer that $\lambda_{21}<0$, λ_71_*>0*. Given that the purpose of this paper is to study the promotion effect of farmland protection assessment mechanisms on township governments and village collectives, if *M*_2_ (1,0,0)is the ideal stable equilibrium point, the condition $p({r_{2}+l}_{2})\gg c_{2}-c_{3}$, This means that the administrative incentives and avoided accountability losses obtained when township governments choose a transparent supervision strategy are much higher than the management costs of concealing the truth. At this time, $\lambda_{22}<0,$λ_32_*>0*. Conversely, if $p({r_{2}+l}_{2})<c_{2}-c_{3,}$then when $r_{4}-pl_{3}-c_{6}{-c}_{5}-r_{3}+c_{4}<0$, the equilibrium point *M*_3_ (1,1,0) will be an evolutionary stable point. In this case, township governments choose to conceal the truth, causing a waste of public resources, which is not the most ideal stable strategy combination. Since $r_{4}-c_{5}-l_{3}<r_{3}-c_{4}$, it can be observed that when the township governments implement transparent supervision, the shift of the village collective from neglecting responsibilities to actively fulfilling them results in an increase in benefits, from $s+r_{4}-c_{5}-l_{3}$ to $s+r_{3}-c_{4}$. This indicates that transparent supervision is the optimal strategy for the township government. Additionally, since $\lambda_{23}<0$, it can be concluded that *M*_2_ (1,0,0) is a stable point. In this scenario, the municipal governments adopt an assessment policy, the township governments enforce strict supervision with transparent monitoring and truthful reporting, and the village collectives actively participate in farmland protection. Based on the above conditions, it can be concluded that *M*_3_ (1,1,0), *M*_4_ (1,0,1) and *M*_7_ (0,0,1) are not stable equilibrium points. The assessment system and the enthusiasm of village collectives in fulfilling their responsibilities are crucial for the stability of the tripartite game strategy. If village collectives lack the motivation to fulfill their responsibilities, even strict supervision by township governments will not lead to evolutionary strategy stability. Furthermore, the absence of an assessment system, due to the lack of corresponding incentives and constraints, directly undermines the administrative credibility of the governments and indirectly weakens the village collectives’ commitment to actively fulfilling their responsibilities.

Second, from the assumption $c_{1}<l_{1}<g$, we can infer that $p(g-l_{1})-c_{1}+2l_{1}$>0, thus *λ*_*51*_*>0、λ*_*61*_*>0、λ*_*81*_<0. It can be judged that the equilibrium points *M*_*5*_ (0,1,0)和*M*_6_ (0,1,1)are unstable, but the stability of the equilibrium point *M*_8_ (1,1,1)still requires additional conditions, which can be proven by contradiction.

Assuming there are two stable equilibrium points, *M*_2_ (1,0,0) and *M*_8_ (1,1,1), the following conditions must be met:


$$p(l_{2}{{-l}_{3}+r}_{2})-c_{6}+c_{3}+l_{3}-c_{2}<0$$ (21)

$$-r_{4}+pl_{3}{+c_{6}{+c}_{5}+r}_{3}{-c}_{4}<0$$ (22)

$$-p(r_{2}+l_{2})+c_{2}-c_{3}<0$$ (23)

$$r_{4}-c_{5}-l_{3}-r_{3}+c_{4}<0$$ (24)

Combining (21) and (23) gives: $c_{2}{-c}_{3}<p(r_{2}+l_{2})<c_{2}{-c}_{3}+(p-1)l_{3}+c_{6}$

Combining (22) and (24) gives: $(p-1)l_{3}+c_{6}<0$, the system of equations is clearly inconsistent. Therefore, the equilibrium point *M*_8_ (1,1,1) is unstable, indicating that the assessment mechanism can promote strict supervision by township governments.

## 4. Simulation results analysis

As mentioned above, in the case of the equilibrium point *M*_2_ (1,0,0), the implementation of the farmland protection assessment system by county governments, transparent supervision by township governments, and active participation by village collectives represents the ideal Evolutionarily Stable Strategy (ESS) for the evolutionary game model of farmland protection assessment system. The implementation costs and benefits of village policies, the financial support from township governments to village collectives, and the administrative rewards and penalties received by township governments all determine the system’s final stable equilibrium state To further explore the dynamic evolutionary paths of the game and identify strategy combinations that achieve stable conditions by optimizing the parameters at the equilibrium point, this paper uses MATLAB R2022b software for simulation. The simulation analyzes the impact of different farmland protection costs and benefits on the strategy choices of the three parties and explores incentive-constraint strategies to enhance the policy implementation enthusiasm of township governments and village collectives.

### 4.1 Parameter assignment

The assignment of model variables and parameters must adhere to economic assumptions and empirical judgments. Due to regional differences in farmland area, subsidy standards per acre vary. For instance, the subsidy standard in Heilongjiang Province is 75.16 RMB per acre, while in Guizhou Province, it is generally no less than 48.45 RMB. In Shandong Province, the subsidy is set at no less than 120 RMB. Zhejiang Province provides an annual subsidy of 30 RMB based on the area of permanent basic farmland protection, which is linked to the assessment of farmland protection responsibility targets and cases of illegal land use. The administrative reward and punishment indicators for township governments are determined based on the “Notice of the State Council on Reforming the Civil Servant Salary System,” which uses the wage differences between the higher and lower salary levels for township-level civil servants (grades 9–12) as a reference. The constraint indicators for village collectives are primarily based on existing laws. Article 75 of the “Land Administration Law” explicitly states that individuals who damage farmland will face fines or criminal penalties. The “Regulations on the Implementation of the Land Administration Law” specify that the fine amount is “5 to 10 times the cost of land reclamation.” Additionally, the “Civil Code” and the “Environmental Protection Law” clearly define the legal responsibilities of those who harm the ecological environment. The assignment of other indicators is based on the previously established relationships between profit and loss variables and their corresponding parameters. The initial values are assigned as follows:

(1) The implementation costs for the complete behaviors of the three parties in the game are set as $c_{4}>c_{1}>c_{2}$, The assessment cost for county governments adopting the assessment strategy is $c_{1}=30$. The supervision costs for township governments under transparent and concealed supervision are $c_{2}=20$ and $c_{3}=5$, respectively. The input cost for the village collectives actively implementing farmland protection is $c_{4}=80$, the input cost for passive implementation is $c_{5}=30$, and the rent-seeking cost is $c_{6}=10$.
(2) As China’s farmland protection policies become more stringent, policymakers typically set multiple indicators to constrain or evaluate the administrative level of governments. The credibility of county governments under strict assessment is set at g=80, while the credibility under failed assessment is $l_{1}=40$, with the public loss caused by this failure being d=150. The initial success rate of the assessment is set at p=0.5. The administrative loss for township governments failing to supervise is $l_{2}=150$, including the loss of administrative rewards and accountability from higher authorities. The accountability loss for the village collectives’ passive performance is $l_{3}=100$.
(3) Farmland is a valuable and scarce resource, and actively implementing farmland protection can bring multiple benefits, including environmental improvement and economic growth. Due to the spillover effects of farmland’s ecological benefits, which affect actual value measurements, this paper sets the initial value of the comprehensive benefits from farmland protection for county governments at $r_{1}=200$. Using the salary increment obtained from the promotion of government staff as the standard for administrative rewards, the administrative rewards for township governments are set at $r_{2}=120$. The village collectives, as the implementers of farmland protection, enjoy direct comprehensive benefits from this protection, set at $r_{3}=180$. The financial support they receive for farmland protection is set at $s=c_{4}=80$, and the speculative gains obtained under passive performance are $r_{4}=120$.

### 4.2 Simulation results analysis

**Fig 2:**
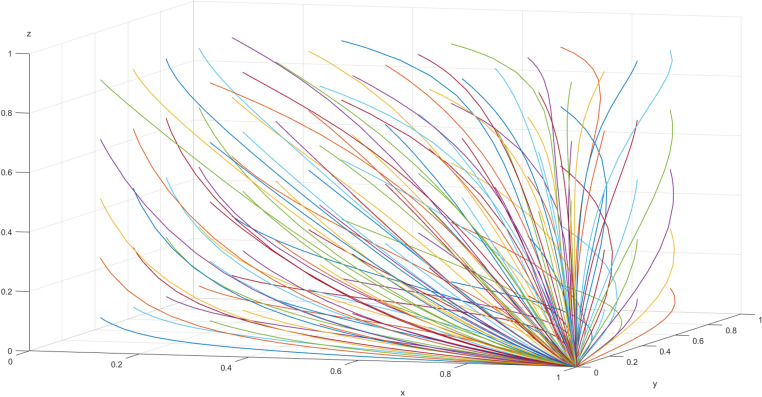
Simulation diagram of the three-party game stable point.

From the simulation results in Fig 2, it can be observed that the system has only one evolutionary stable strategy combination, *M*_2_ (1,0,0) (assessment, transparent supervision, active performance), which is consistent with the conclusions above.

**Fig 3:**
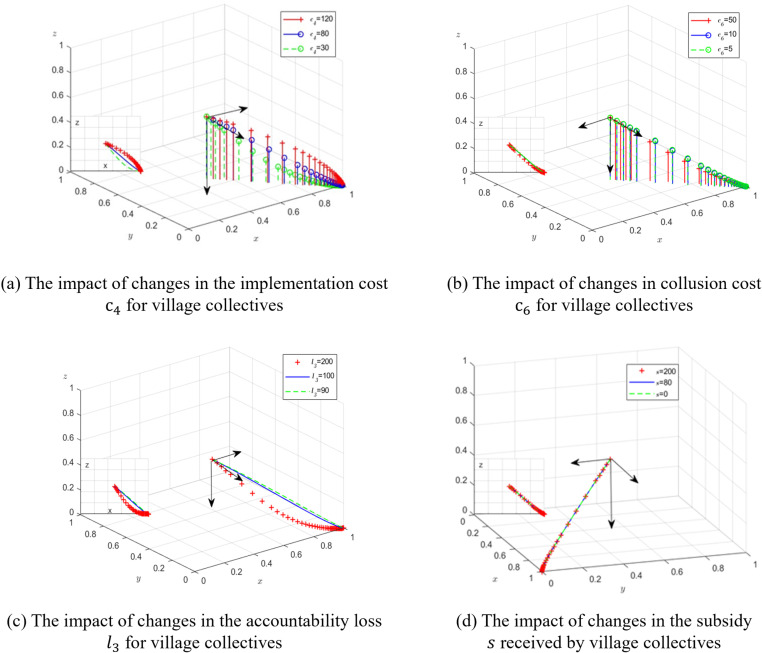
Schematic diagram of the evolutionary process of the three parties under changes in village collectives’ costs and benefits.

The policy implementation costs and opportunity costs of village collectives directly affect the level of grassroots farmland protection investment. These costs mainly stem from the administrative cost of policy implementation ($c_{4}$), the collusion cost paid to township governments ($c_{6}$), and the accountability loss when discovered $l_{3}$. To explore the impact of changes in the administrative cost $c_{4}$ on the evolutionary game when village collectives actively perform their duties, the values are set to $c_{4}=30,80,and120$. The simulation results of the dynamic equations, evolved over 50 iterations, show that as $c_{4}$ increases, the evolutionary speed of village collectives tending to actively perform their duties slows down. Fig 3a and 3b show that during the system’s evolution to the stable point, an increase in collusion cost $c_{6}$ slows down the evolutionary speed of village collectives actively implementing farmland protection. As the collusion cost increases, the probability of village collectives actively performing their duties rises, while the probability of transparent supervision by township governments decreases. County governments can raise the collusion cost for village collectives through grassroots self-governance measures, such as mutual monitoring and evaluation within the town, incorporating these into branch evaluations, thereby increasing the probability of active farmland protection. To analyze the impact of changes in accountability loss $l_{3}$ on the evolutionary game process and results, the values are set to $l_{3}=90,100,and200$. The simulation results of the dynamic equations, evolved over 50 iterations, are shown in Fig 3c. During the evolution process, as the accountability loss $l_{3}$ increases, the probability of county governments regularly assessing grassroots farmland protection performance and issuing administrative rewards decreases, while the probability of township governments concealing the truth also decreases.

The farmland protection benefits for village collectives consist of direct comprehensive benefits, financial support, and indirect speculative gains. These include the comprehensive farmland benefits, financial support provided by township governments, and speculative gains of village collectives. The comprehensive benefits are difficult to measure and primarily include the ecological and societal benefits resulting from the implementation of farmland protection policies. Indirect speculative gains only occur in the non-ideal situation of passive performance. Therefore, this paper focuses on examining the impact of financial support provided by township governments on the strategy choices of farmland protection subjects. In this analysis, the financial support is set to s=0,80,and200, while other parameters remain unchanged. The simulation results of the dynamic equations, evolved over 50 iterations, are shown in Fig 3d. During the system’s evolution to the stable point, the level of financial support provided by the township governments to the village collectives decreases, but this does not affect the village collective s ‘ strategic choice or the time it takes for the three parties to reach a stable equilibrium. Therefore, regardless of whether the township governments provide financial support to the village collective, the latter are more likely to adopt an active farmland protection strategy.

**Fig 4:**
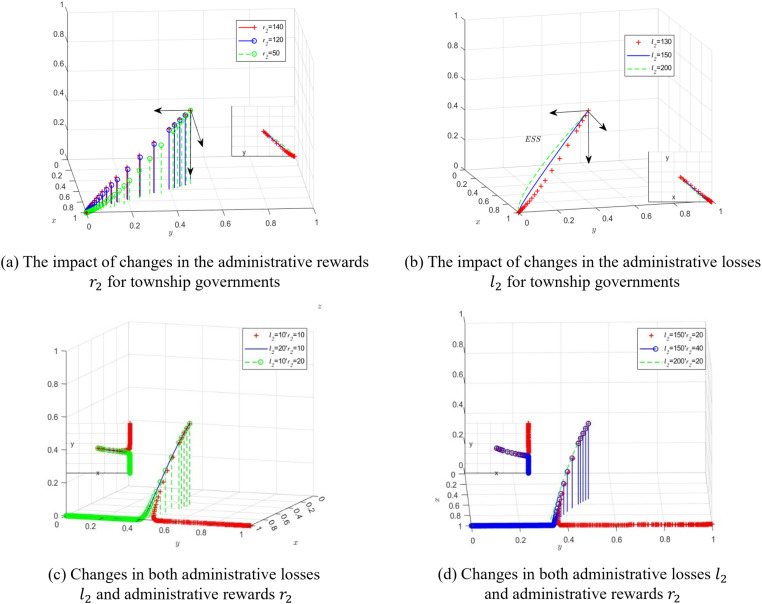
Schematic diagram of the evolutionary process of the three parties under.

In that case, if county governments adopt an assessment strategy for township governments, can it optimize the overall implementation of farmland protection policies? If the assessment strategy is effective, should the incentive-constraint mechanism linked to the assessment focus more on punishment or rewards? And what standards should it follow? To answer to these questions, further system simulations are conducted as follows:

First, software simulation is used to model the impact of changes in the level of administrative rewards (incentives) and administrative losses (constraints) on the strategy choices of the three parties. The administrative rewards received by township governments are assigned values of $r_{2}=50,120,and14$0, and the simulation results of the dynamic equations over 50 iterations are shown in Fig 4a. As $r_{2}$ increases, the administrative rewards for strict supervision by township governments also increase, reducing the probability of concealing the passive performance of village collectives and decreasing the likelihood of passive performance by village collectives. Next, the impact of changes in administrative losses on the strategy choices of the three parties is simulated. The administrative losses of township governments when discovered are assigned values of $l_{2}=130,150,and200$, and the simulation results of the dynamic equations over 50 iterations are shown in Fig 4b. As the administrative losses of township governments increase, the probability of concealing the passive performance of village collectives decreases, while the probability of passive performance by village collectives increases.

The success rate of county governments’ assessments also affects the strategy choices of township governments, thereby impacting the quality of farmland protection by village collectives. Therefore, while keeping other factors unchanged, this study explores whether administrative rewards (incentives) or administrative losses (constraints) are more effective for township governments under high or low success rates of county governments’ assessments. When the success rate of assessment is high (p=0.6), the probability of discovering township governments’ concealment of the truth and village collectives’ passive performance increases, thereby strengthening the self-supervision and self-restraint awareness of the policy implementation subjects. During the simulation, the values are assigned as follows: $p=0.6,l_{2}=10,r_{2}=10$*;* $p=0.6,l_{2}=20,r_{2}=10$ and $p=0.6,l_{2}=10,r_{2}=20$. The simulation results of the dynamic equations over 50 iterations are shown in Fig 4c. The results show that the strategy combination *M*_3_ (1,1,0) only appears when both the incentive and punishment levels are very low. Increasing any of the incentive or punishment levels shifts the game results toward the ideal state of *M*_2_ (1,0,0). Moreover, as the success rate of assessments increases, the rate at which township governments and village collectives adopt transparent supervision and actively fulfill their responsibilities accelerates. Therefore, when the success rate of county governments’ assessment is high, it can fully mobilize township governments and village collectives to actively implement farmland protection policies, which helps save societal resources.

When the success rate of assessment is low (*p*=0.08), the risk of being penalized for the implementation subjects is relatively weakened, and the tolerance level for punishment increases. Under high-level administrative punishment and low-level administrative rewards, the values are assigned as follows: $p=0.08,l_{2}=150,r_{2}=20$*;* $p=0.08,l_{2}=200,r_{2}=20$*;* $p=0.08,l_{2}=150,r_{2}=40$, and the simulation is run. The simulation results of the dynamic equations over 50 iterations show that when the punishment is 7.5 times the incentive ($l_{2}=150,r_{2}=20$), the equilibrium point of the evolutionary game eventually tends to *M*_3_ (1,1,0), meaning township governments would rather “take risks” and be penalized. Township governments will choose to diligently perform their duties only when the punishment increases to 10 times the incentive ($l_{2}=200,r_{2}=20$). When the incentive slightly increases to 40 ($l_{2}=150,r_{2}=40$), with the punishment being 3.75 times the incentive, the equilibrium point of the evolutionary game eventually shifts to *M*_2_ (1,0,0), indicating that township governments are more inclined to choose a lower level of reward rather than risk being penalized. Clearly, township governments are more sensitive to administrative rewards, as shown in Fig 4d.

**Fig 5:**
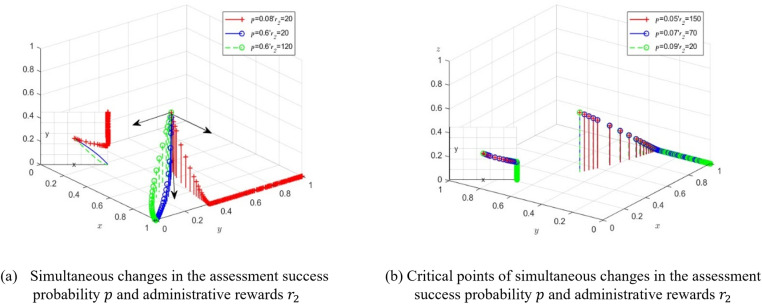
Schematic diagram of the evolutionary process of the three parties under changes in administrative reward thresholds and incentive assessment success probability.

Furthermore, while keeping the initial values of other factors unchanged, we examine the strategy choices of the three parties under simultaneous changes in the county governments’ assessment success probability (p) and the administrative rewards$\left(r_{2}\right)$ received by township governments. The values and simulation results are shown in Fig 5a. We find that when p=0.6, $r_{2}=20$, and $p=0.6,r_{2}=120$, that is, when the county governments’ assessment success probability is high, the level of administrative rewards does not affect the strategy preference for transparent supervision by township governments. As the administrative rewards ($r_{2}$) increase, the system reaches the stable equilibrium point *M*_2_ (1,0,0) more quickly. However, when $p=0.08,r_{2}=20$, that is, when county governments’ assessment success probability is low, the system’s final stable equilibrium point is *M*_3_ (1,1,0). At this point, township governments may conceal the truth in order to receive rewards, leading to a waste of societal public resources and negatively affecting the formation of government credibility.

So, when the assessment success probability is low, can the strategy combination be optimized by appropriately increasing administrative rewards? To explore this, we keep the initial values of other factors unchanged and only vary the assessment success probability p and administrative rewards $r_{2}$. Under the low success rate of assessment, the values are assigned as p=0.05,0.07,0.09, and the corresponding critical values of administrative rewards for the system to reach the stable equilibrium point *M*_2_ (1,0,0)are $r_{2}=150,70,20$ respectively. It can be seen that when county governments’ assessment success rate is low, a higher level of administrative rewards is needed to promote the joint implementation of policies by multiple subjects. According to the principle that the economic benefits of policy implementation should be higher than the policy input costs, the level of administrative rewards should be at least higher than the administrative costs of transparent supervision, as shown in Fig 5b.

### 4.3 Institutional implications

The theoretical derivation, mathematical calculations, and simulation analysis presented in this paper lead to the following policy recommendations, aiming to provide effective theoretical guidance and practical references for optimizing the implementation of farmland protection policies.

To ensure the effective implementation of farmland protection policies, it is crucial to address key issues such as information asymmetry and the design of incentives and constraints within the principal-agent framework. In this context, county governments, as the principal, directly influences the actions of township governments and village collectives through its assessment mechanisms. A high assessment success rate allows county governments to implement administrative rewards and penalties effectively, thereby enhancing the accountability and motivation of all parties involved and promoting collaborative efforts. However, if the assessment system is flawed or inefficient, it may lead to opportunistic behavior among township governments and village collectives, undermining the overall effectiveness of policy implementation. Therefore, it is essential to improve the efficiency of the assessment process and ensure that incentive and constraint measures are properly adjusted to reflect changes in the execution environment, optimizing the policy’s impact.

The assessment mechanism in policy implementation exhibits a two-way feedback effect. The execution capacity of township governments is influenced by two factors: firstly, the assessments conducted by county governments, and secondly, the supervisory actions and performance of township governments, which in turn can influence the decisions of county governments. In this context, county governments must be able to adjust its incentive and constraint measures flexibly to ensure the sustainability and effectiveness of policy implementation. To this end, county governments should enhance information transparency, establish an open assessment system, and introduce third-party evaluations to ensure fairness and transparency in assessments, thereby preventing adverse selection and moral hazard. However, in the context of limited fiscal resources, reliance on administrative penalties alone is unlikely to yield optimal results. Instead, increasing administrative rewards and other incentive mechanisms can more effectively motivate township governments to fulfil their responsibilities, improve regulatory transparency, and indirectly help village collectives address issues such as insufficient funding or excessive investment. Furthermore, the introduction of reward measures has been demonstrated to be more efficacious than reliance on punishment alone in enhancing the execution capacity of regions with low assessment success rates and encouraging behavioral improvement. This mechanism fosters a reciprocal relationship between fiscal resources and performance, thereby enhancing the overall effectiveness of the policy.

The efficacy of farmland protection is contingent not only on the appropriate implementation of policies, but also on the judicious allocation of responsibilities and benefits, thereby ensuring comprehensive cooperation among all relevant parties. Consequently, the establishment of a comprehensive farmland protection system is imperative, encompassing long-term monitoring mechanisms and incentives for voluntary participation, whilst implementing differentiated rewards according to responsibilities at various levels and conducting layered performance assessments. This mechanism is pivotal in ensuring long-term cooperation among all stakeholders in the protection of farmland, thereby preventing the evasion of responsibilities driven by short-term interests. In addition, township governments should proactively seek financial support to enable them to assume responsibility for farmland protection. They should establish a stable financial support and reward system in collaboration with village collectives. In circumstances where fiscal resources are constrained, the judicious allocation of rewards and incentives can foster enhanced transparency in supervision, thereby aiding village collectives in surmounting financial constraints and ameliorating funding shortfalls in policy implementation. The establishment of such a stable financial support system is therefore recommended, as it would serve to strengthen the motivation of township governments and village collectives in fulfilling their responsibilities, thereby driving the long-term and stable implementation of farmland protection policies.

## 5. Conclusions

This paper constructs a three-party evolutionary game model of farmland protection in China, considering factors such as government credibility, the comprehensive benefits of farmland protection, the administrative incentives of county governments, administrative costs for township governments, and implementation costs for village collectives. The study provides a comprehensive examination of the evolutionary paths of strategy choices, the migration of stable points, and evolutionary rates for county governments, township governments, and village collectives. The research’s findings offer valuable guidance for the enhancement of implementation strategies and systems of farmland protection at these levels. The research findings indicate that the current farmland protection evaluation system is deficient in its oversight of the responsibilities of township governments in implementing farmland protection, and that it lacks effective administrative incentives and constraints. There is an urgent need to link the administrative incentives for township governments to their performance in farmland protection, integrating the incentive mechanism systematically into the evaluation framework. This integration would facilitate a direct correlation between incentives and performance assessments, thereby incentivising township governments to fulfil their responsibilities.

It is imperative that the constraint mechanism is closely aligned with the evaluation system. Furthermore, it is important to note that incentives and constraints are not independent entities; rather, they are interdependent, and their combination demonstrates varying effectiveness in different contexts. The role of administrative incentives and constraints varies across regions, and the government should tailor incentive and constraint policies according to regional differences. In regions with high evaluation success rates, administrative incentives can effectively enhance the enthusiasm of all parties involved, stimulating the participation of township governments and village collectives. The strength of administrative rewards should be sufficient to outweigh administrative costs. In regions with inefficient evaluation mechanisms or low success rates, the impact of incentives alone is limited. In such cases, relying solely on incentives may not ensure the smooth implementation of policies, and administrative constraints must be strengthened. In areas with relatively tight fiscal resources, county governments can optimize the combination of incentives and constraints. For instance, while providing rewards, they should also increase penalties for township governments that fail to perform adequately, creating a synergistic effect between positive incentives and negative constraints. Where necessary, county governments can offer additional fiscal subsidies or tax incentives to encourage township governments to improve transparency and effectiveness in farmland protection. In economically developed areas, increasing the intensity of supervision and penalties can help ensure that policy responsibilities are fulfilled without complacency. It is incumbent upon county governments to establish transparent evaluation and feedback mechanisms, ensuring that the performance of township governments is promptly reflected on public platforms, subject to societal oversight. Furthermore, the establishment of a performance-based accountability mechanism is imperative to facilitate the swift rectification of any deviations in behavior.

The farmland protection system is of pivotal importance for China’s food and ecological security, and is also an indispensable component of the global food production and supply chain. The “trinity” farmland protection system is of significant theoretical value and practical importance, as it involves the optimisation of policies for county governments and township governments by exploring the key factors and benefit connections in the farmland protection decisions of township governments and village collectives. This optimisation could help to balance the fiscal pressures and administrative inertia of township governments. Future research could further explore areas such as cost-sharing between township governments and village collectives, as well as the feasibility of compensating farmers for their participation. Such discourse would provide both theoretical underpinnings and practical guidance for enhancing the system and ensuring the effective implementation of policies.
